# A comparative study of the efficiency and safety of chemotherapy as a therapeutic method for recurrent or resistant gestational trophoblastic neoplasia: A protocol for systematic review and meta-analysis

**DOI:** 10.1097/MD.0000000000026263

**Published:** 2021-06-18

**Authors:** Tian-Yue Jiang, Jia-Jie Ren, Yang Zhang, Yan Zhao, Xiao-Ling Feng

**Affiliations:** Department of Obstetrics and Gynecology, the First Affiliated Hospital of Heilongjiang University of Chinese Medicine, Harbin, Heilongjiang, China.

**Keywords:** chemotherapy, efficiency, gestational trophoblastic neoplasia, resistant

## Abstract

**Background:**

Gestational trophoblastic neoplasia (GTN) is an infrequent spectrum of placental malignant cases. Generally, single-agent or multiple-agent chemotherapy is used to treat the condition. The condition has a significant impact on women in the childbearing age, which makes post-chemo fertility and obstetrical results a significant contemplation. Nearly 25% of GTN tumors are recurrent, or have a likelihood of relapsing after, the first round of chemotherapy. Therefore, these resistive and recurring lesions require salvage chemotherapy with or without surgical treatment. Therefore, the current meta-analysis and systematic review will assess the effectiveness and level of safety when using chemotherapy to treat women with resistive or recurring GTN.

**Methods:**

The current study will perform a comprehensive systematic search for randomized controlled trials (RCTs) that have assessed the efficacy and safeness of chemo as a line of treatment for women with resistive or recurring GTN. To this end, a search will be conducted on the following electronic databases: Web of Science, MEDLINE, Chinese National Knowledge Infrastructure (CNKI), EMBASE, WanFang database, and the Cochrane Library. The search will cover the period from the inception of databases to May 2021. In order to identify additional related studies, we will manually search the reference lists of suitable research articles and related systematic reviews. A pair of independent authors will review the titles/abstracts of the studies to check if the studies are eligible, which is proceeded by screening the full texts. This study will employ a uniform data extraction table for data extraction. Moreover, based on the Cochrane Risk of Bias Tool, this protocol review also assesses the bias risk in the studies involved.

**Results:**

A comprehensive synthesis of existing indication on chemo treatment for women with resistive or recurring GTN.

**Conclusion:**

The results offer fresh references for evaluating the effectiveness and safeness of chemo-based treatment for women with resistive or recurring GTN.

**Ethics and dissemination:**

An ethical approval is not needed as all data are published.

**Review registration number:**

May 17, 2021.osf.io/uwky7. (https://osf.io/uwky7/).

## 1 Introduction

Gestational trophoblastic neoplasia (GTN) characterizes a spectrum of rarely occurring malignancies in the placenta, encompassing tenacious gestational trophoblastic condition, aggressive moles, choriocarcinoma, and both trophoblastic and epithelioid trophoblastic tumors centered around the placenta.^[1]^ Considering the fact that most women diagnosed with GTN are of gestation age, preservation of fertility is a vital consideration.^[1]^ Generally, GTN is prevalent after molar pregnancies, but could also occur after any antecedent pregnancy.^[2]^ Fully developed moles are paternally derived and contain a diploid karyotype, while partial moles tend to be triploid, forming 2 sets of haploid genes, maternally and paternally.^[2,3]^ Generally, molar pregnancies resolve impulsively after a single uterine evacuation, or even >1, with chemotherapy or otherwise. Still, the disease prevails in nearly 20% of complete moles and 1% of partial moles, advancing to GTN, which necessitates chemotherapy.^[2,4–6]^

Chemotherapy is the first line of treatment for GTN. The choice of getting single-agent and multi-agent chemo depends on the prognostic score of the International Federation of Gynecology and Obstetrics.^[7]^ Generally, women who fail to score 7 are characterized as low-risk and single-agent chemotherapy is the preferred mode of treatment, typically comprising actinomycin-D or methotrexate, where the overall cure rate is almost 100%.^[7]^ In persistent cases, treatment can be switched to actinomycin-D or multi-agent chemotherapy.^[7,8]^ In most instances, high-risk disease is treated with a regime of chemotherapy involving methotrexate, etoposide, cyclophosphamide, actinomycin-D, and vincristine. In resistive or recurring cases, the secondary form of chemotherapy comprising paclitaxel/etoposide alternated with paclitaxel/cisplatin or bleomycin is adopted widely.^[9,10]^ Reportedly, the complete cure rate for high-risk cases is 94%.^[11]^ Thus, treating resistive or recurring GTN is still a challenge and has increasing epidemiological relevance for global women. Presently, chemotherapy is approved for women with recurring or resistive GTN. Therefore, it is imperative to gain a good understanding about the efficacy and safety of chemotherapy treatment for women with resistive or recurring GTN. The present study evaluates the efficacy and safeness of using chemotherapy as a first line of treatment for women with resistive or recurring GTN.

## 2 Methods

### 2.1 Study registration

The design of the present study adheres to the Preferred Reporting Items for Systematic review and Meta-Analysis Protocol (PRISMA-P) statement, and it is registered in OSF (https://osf.io/) with 10.17605/OSF.IO/UWKY7.^[12]^

### 2.2 Criteria for including studies

#### 2.2.1 Types of studies

This comparative study includes all randomized controlled trials (RCTs) assessing the effectiveness and safeness of using chemo to treat women with resistive or recurring GTN. The present review only includes studies published in English and Chinese.

#### 2.2.2 Types of participants

Women diagnosed with GTN shall be included. There are no restrictions on the age and ethnicity of participants.

#### 2.2.3 Types of interventions

Any chemotherapy regime used as treatment for recurring or resistive GTN is considered, the chemo has no restrictions on dosage, frequency, duration, or combination.

#### 2.2.4 Types of outcome measures

The primary outcomes include entire response rate, remission rate, and overall prognosis for surviving. The secondary outcomes include the mean number of courses required for curing, average number of courses to failure, secondary cancers, and toxicity grades based on CTCAE 2010, including hematological, gastrointestinal, genitourinary, neurological, and respiratory.^[13]^

### 2.3 Search methods for identifications of studies

#### 2.3.1 Electronic searches

The current study will perform a comprehensive systematic search for RCTs that have evaluated the effectiveness and safeness of chemotherapy for treating women with resistive or recurring gestational trophoblastic neoplasia (GTN). To this end, a search will be conducted on the following electronic databases: Web of Science, MEDLINE, Chinese National Knowledge Infrastructure (CNKI), EMBASE, WanFang database, and the Cochrane Library. The search will cover the period from the inception of databases to May 2021. Afterwards, the Boolean operators “OR” and “AND” will be used to combine the following search terms: GTN, chemotherapy, randomized controlled trial.

#### 2.3.2 Electronic searches

The authors will also search clinical trials (www.clinicaltrials.gov) to identify incomplete trials. Moreover, we will perform a search manually in the reference lists of suitable research articles and related systematic reviews to recognize additional related research articles.

### 2.4 Data collection and analysis

#### 2.4.1 Study selection

**Figure 1:**
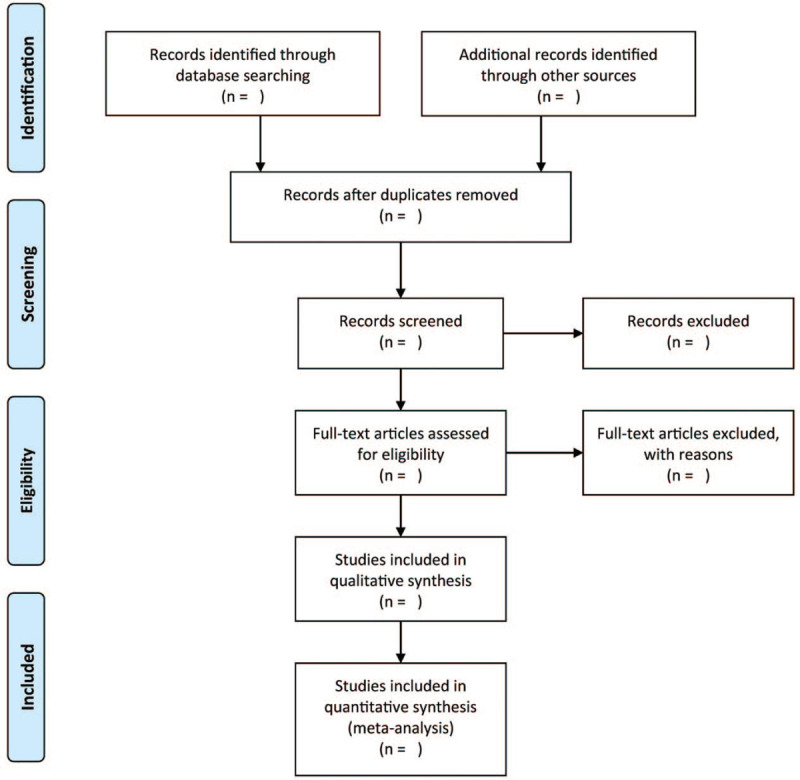
The research flowchart.

A pair of independent authors will analyze the headings/summaries of the studies to identify eligible studies, which is proceeded by screening of the full texts. All disagreements are mediated via discussion with another independent author. The reasons for rejecting studies will be recorded and reported in the flowchart following full-text screening. Figure 1 illustrates the flowchart outlining the process of selecting studies.

#### 2.4.2 Data extraction and management

A pair of autonomous authors will perform data extraction using a pre-designed data collection form. The data contain basic info (first author, country, published day, and ethnicity), study design (methodology, intervention chemotherapy regime, dosage, frequency, duration, and treatment period), and outcome measures. All disagreements will be mediated through discussion with another independent author.

#### 2.4.3 Assessment of risk bias

Based on the Cochrane Collaboration Tool, we will evaluate the bias risk in the selected RCTs.^[14]^ All differences in opinion shall be fixed via discussion with a third autonomous author.

#### 2.4.4 Measures of treatment effect

For time-to-event data, this protocol report will utilize the hazard ratio (HR) for comparing the risk of mortality or progression of the condition in the experimental group with that in the control group. For dichotomous outcomes, the relative risk (RR) will be used for analysis. In each estimate, the 95% confidence intervals (CIs) will be reported.

#### 2.4.5 Assessment of heterogeneity

The *X*^2^ test and *I*^2^ statistic shall be utilized to assess the heterogeneity among the articles. The random-effects models will be adopted if *I*^2^ > 50% and *P* < .1; otherwise, the fixed-effects model will be utilized.^[15,16]^

#### 2.4.6 Assessment of reporting biases

All instances of reporting bias shall be assessed using funnel plots.

#### 2.4.7 Sensitivity analysis

It is intended to exclude studies having high bias risk to investigate the impact on the combined results.

## 3 Discussion

Presently, no meta-analysis and systematic review have investigated the effectiveness and safety level of using chemotherapy to treat women with resistive or recurring GTN. The present protocol review explores this issue comprehensively and we will conduct a complete literature review of all articles published in English and Chinese to identify additional related studies. All potential studies related to assessing the safety and effectiveness of using chemotherapy to treat women with resistive or recurring GTN will be included. The work done will provide an updated summary of assessing the efficacy and safeness of chemo when used as a healing strategy for women with resistive or recurring GTN. The results will provide evidence that helps decide whether chemotherapy is effective and safe at curing recurring or resistive GTN in women. Moreover, the evidence could also provide useful evidence for clinical practitioners and health policymakers.

## Author contributions

**Conceptualization:** Tian-Yue Jiang, Yang Zhang.

**Data curation:** Tian-Yue Jiang.

**Formal analysis:** Tian-Yue Jiang, Xiao-Ling Feng.

**Funding acquisition:** Jia-Jie Ren, Yang Zhang, Xiao-Ling Feng.

**Investigation:** Tian-Yue Jiang, Yan Zhao.

**Methodology:** Yang Zhang, Yan Zhao.

**Resources:** Jia-Jie Ren, Yang Zhang, Yan Zhao, Xiao-Ling Feng.

**Software:** Tian-Yue Jiang, Jia-Jie Ren, Xiao-Ling Feng.

**Supervision:** Jia-Jie Ren, Yang Zhang.

**Validation:** Tian-Yue Jiang, Yan Zhao.

**Visualization:** Tian-Yue Jiang.

**Writing – original draft:** Tian-Yue Jiang.

**Writing – review & editing:** Yan Zhao, Xiao-Ling Feng.
